# Supplementary material for: Violence against women on Twitter in India: Testing a taxonomy for online misogyny and measuring its prevalence during COVID-19
Source: PLoS One. 2023 Oct 25;18(10):e0292121. doi: 10.1371/journal.pone.0292121 (PMC10599529; doi:10.1371/journal.pone.0292121)
Supplement: S1 File — (PDF) [file pone.0292121.s001.pdf]

S1 File. Annotation guidelines/manual for undergraduate student coders

The purpose of this annotation is to classify a group of tweets from India as misogynistic. These posts have been randomly selected from a larger group of tweets that were posted from India. Each tweet will be annotated by at least two coders separately. The final product from this exercise (classified tweets) will be used to predict misogynistic content across a large dataset of geotagged tweets (over 30 million tweets) posted from India between 2018 and 2021, using machine learning models.

This document is intended to guide annotators in classifying the tweets as misogynistic/non-misogynistic.

The annotation exercise will be carried out using google sheets. Only assigned annotators and the researchers involved in the study will have 'editing' access to the sheets. Each row in the google sheet corresponds to a single tweet. Annotators will answer the specified questions for each tweet, or fill columns C-G in the google sheet, for each tweet. The responses to all questions are Yes, and No, to be noted as 1 (for Yes) and 0 (for No) in the sheet. There are four questions for each tweet, with the first three questions being screening questions that identify whether the tweets have comprehensible content. The questions are:

1. Does the tweet contain language that you are unable to interpret or understand (words typed in English but possibly in a different language, or too many typos)?

   1= Yes → If YES, start answering questions for next tweet.

   0= No → Go to next question for same tweet

2. Does the tweet contain images or links, and they are necessary to understand the meaning of the tweet?

   1= Yes → If YES, start answering questions for next tweet.

   0= No → Go to next question for same tweet

3. Does the tweet contain figurative text that makes the intention or meaning of the text unclear?

   1= Yes → If YES, start answering questions for next tweet.

   0= No → Go to next question for same tweet

If the response to all three questions are 'No', the coder will assess whether the tweet contains misogynistic content, and answer question 4:

4. Does this tweet contain hate speech against women defined as "*rhetoric or content that uses hostile and malicious language targeted at women, objectifies women, threatens women with physical or sexual harm, tries to ensure the inferiority of women, justifies gender-based violence, or discredits feminist activists and their efforts*".

   1= Yes

0= No → If NO, start answering questions for next tweet.

Identifying misogyny in tweets: We define misogyny as any rhetoric or content that uses hostile and malicious language targeted at women, objectifies women, threatens women with physical or sexual harm, tries to ensure the inferiority of women, justifies gender-based violence, or discredits feminist activists and their efforts. The tweet contains misogynistic speech if slurs or derogatory language are used toward any woman, or group of women, regardless of whether or not the target is a specific person. (e.g. a tweet without a direct target: "*You f***ing sl***" still contains misogynist speech). Tweets that do not include any slur words, but try to enforce the inferiority of women, or discredit feminist movements will also be classified as misogyny. Additionally, certain tweets that dehumanize women (most often in a sexual context/pornographic content) will be categorized as misogynist. The following table covers a few examples for different types of tweets that can or cannot be classified as misogyny. All examples are real tweets posted in 2020.

**Tweet**	**Classification**
You seriously are slut. There is no doubt on that.	Misogyny
Bitch please.	Not misogyny – while this tweet contains a slur word, the context of its use is unclear.
Get that bitch behind the bar.	Misogyny

I said it. SLUT. BITCH. and all those words that are offensive to her.	
Dirty fucking Muslim democrats desecrated tulsi sacred plant in a Hindu house. Shame on these bastards. Shame on Biden and his whore.	Misogyny
Fake feminism is more dangerous than Corona. Women safety is important but it doesn't mean you blame innocent boysâ€¦ https://t.co/eF2AePv9V2	Misogyny – this tweet discredits feminist movements
Better to die from Coronavirus than Feminazivirus	Misogyny – this tweet discredits feminist movements
Lets speak about #MenToo . False cases defamed men in a livable society. Many rape lies cases registered daily	Misogyny – this tweet discredits feminist movements, justifies gender based violence
Beautiful pussy	Misogyny – sexual objectification
Hey guy's u ever seen a desi burka slut riding dildo<br>Full video in 15k pkr<br>Just inbox if u want to buy<br>Full video	Misogyny – derogatory and offensive language, and sexual objectification
No. We were slut shamed to extreme lengths by most faculty members if we wore clothes they deemed inappropriate	Not misogyny – although this includes a slur word, the tweet is not hateful

Quality control: Each tweet will be annotated by at least two coders. Separate google sheets will be maintained for each coder to avoid overwriting and mistakes- tweets can be linked across sheets using tweet IDs. The rates of agreement between coders will be examined at the end of the annotation process. Any disagreement between coders will be resolved by the lead researcher.

The tweets contain hateful content. If you (coders) feel triggered or uncomfortable at any point during the exercise and wish to discontinue, please feel free to contact the lead researcher.
