# Supplementary material for: Violence against women on Twitter in India: Testing a taxonomy for online misogyny and measuring its prevalence during COVID-19
Source: PLoS One. 2023 Oct 25;18(10):e0292121. doi: 10.1371/journal.pone.0292121 (PMC10599529; doi:10.1371/journal.pone.0292121)
Supplement: S2 File — (DOCX) [file pone.0292121.s002.docx]

Disclaimer: This file includes words or language that is considered profane, vulgar or offensive by some readers. Due to the topic studied in this article, quoting offensive language is academically justified but we nor PLOS in no way endorse the use of these words or the content of the quotes.

**S2 File. Definition and example tweet for each category of online misogyny**

| **Online misogyny category** | **Definition** | **Example tweets** |
| --- | --- | --- |
| Sexist abusive content | Text that abuses women for no specific reason; contains gendered slur words | *Fuck off bitch* |
| Sexual objectification | Text that sexually objectifies women’s bodies, and treats women’s bodies as objects to be valued for its use by men | *You have a nice pussy* |
| Threatening to harm | Text with threats of physical or sexual harm to women | *I am going to kill you bitch* |
| Asserting women’s inferiority | Text that establishes superiority of men, with an aim to dominate over women | *That’s because women are the weaker sex and people cheer for the underdog usually.* |
| Justifying violence against women | Text that deflects attention from women’s assertion of VAW, and justifies violence | *..had used earlier men for her career enhancement and now also they are crying false rape charges to get money* |
| Dismissing feminist efforts | Text that questions the credibility of feminist activists and the feminist movement | *feminism is more dangerous than Corona!* |
